# A Review of Acute Coronary Syndrome and its Potential Impact on Cognitive Function

**DOI:** 10.5334/gh.934

**Published:** 2021-08-04

**Authors:** Dominika Kasprzak, Janusz Rzeźniczak, Teresa Ganowicz, Tomasz Łuczak, Marek Słomczyński, Jarosław Hiczkiewicz, Paweł Burchardt

**Affiliations:** 1 Department of Cardiology, J. Strus Hospital, ul. Szwajcarska 3, 61-285 Poznań, PL; 2 University of Zielona Góra, ul. Licealna 9, 65-417 Zielona Góra, PL; 3 Department of Cardiology, Nowa Sól Multidisciplinary Hospital, ul. Chałubińskiego 7, 67-100, PL; 4 Department of Hypertension, Angiology, and Internal Medicine, Poznan University of Medical Sciences, 61-848 Poznań, PL

**Keywords:** Cognitive Dysfunction, Dementia, Myocardial Infarction, Acute Coronary Syndrome

## Abstract

According to the World Health Organization (WHO) forecasts, in 2030, the number of people suffering from dementia will reach 82 million people worldwide, representing a huge burden on health and social care systems. Epidemiological data indicates a relationship between coronary heart disease (CHD) and the occurrence of cognitive impairment (CI) and dementia. It is known that both diseases have common risk factors. However, the impact of myocardial infarction (MI) on cognitive function remains controversial and largely unknown. The main goal of this study is to attempt to summarize and discuss selected scientific reports on the causes, mechanisms and effects of CI in patients after acute coronary syndrome (ACS), especially after MI. The risk of CI can increase in patients after ACS, and can therefore also adversely affect the further course of treatment. A late diagnosis of CI can lead to serious clinical implications, such as an increase in the number of hospitalizations and mortality.

## Introduction

The phenomenon of population aging has reached its highest level in world history. According to Eurostat forecasts, the percentage of people aged 65 and older will account for 29.1% of the total population in the European Union by 2080 [1]. Old age is a recognized risk factor for both cardiovascular disease and cognitive impairment (CI) or dementia, which can significantly impair the functioning of those affected. Due to significant health implications and limited therapeutic options, the problem of cognitive dysfunction has become the subject of many studies, even within the field of cardiology. The main aim of this review is to look at the problem of cognitive deficits in patients after acute coronary syndromes (ACS). The available literature is poor in information on this subject, and the mechanisms underlying these disorders remain largely unknown. In addition, much of the data is controversial or inconsistent. Therefore, this review will attempt to summarize and discuss selected scientific reports on the causes, mechanisms and effects of CI in patients after ACS, especially in patients after myocardial infarction (MI), treated with primary coronary artery angioplasty.

## Cognitive dysfunctions

According to ICD-10, dementia is a syndrome due to disease of the brain, usually of a chronic or progressive nature, in which there is disturbance of multiple higher cortical functions, including memory, thinking, orientation, comprehension, calculation, learning capacity, language, and judgement. Consciousness is not clouded. The impairments of cognitive function are commonly accompanied, and occasionally preceded, by deterioration in emotional control, social behavior, or motivation [2]. Dementia is an important global health problem. It is estimated that it affects around 50 million people worldwide, and its incidence increases rapidly with age, and the prevalence rate doubles every 5.9 years. According to WHO forecasts, in 2030, the number of people suffering from dementia will reach 82 million people worldwide, representing a huge burden on health and social care systems [3,4]. That is why it is so important to actively search for risk factors and predisposing conditions in order to recognize its occurrence and raise social awareness as a result. Patients presenting with cognitive deficits that do not significantly affect daily functioning are classified as having mild cognitive impairment (MCI). The prevalence of the latter also increases with age, amounting to 6.7% and 25.2% for people aged 60–64 and 80–84 years old, respectively [5]. People diagnosed with MCI have an increased risk of developing dementia [6,7]. The annual progression rate is estimated to be between 5–10% [8]. There are several types of MCI depending on the type of impaired cognitive function:

- amnestic MCI (a-MCI), in which memory disorders dominate,
- non-amnestic MCI (na-MCI), with impairment of another cognitive function other than memory e.g., language function or visual-spatial function, executive function etc.
- multidomain- MCI, in which more than one cognitive domain is disturbed [9,10].

A-MCI usually leads to Alzheimer’s disease (AD), while patients who do not present with memory deficits are more likely to develop other types of dementia, such as vascular dementia (VaD), frontotemporal dementia, or dementia with Lewy bodies [11,12].

## Cognitive Impairment and Coronary Heart Disease

Epidemiological data indicates a relationship between coronary heart disease (CHD) and the occurrence of CI and dementia [13,14]. However, a limited number of studies have looked at the prevalence of such conditions in patients after MI. The longitudinal study by Roberts et al., involving 1,969 participants aged 70–89, analyzed the relationship between CHD (defined as MI, angina pectoris (AP), angiographic coronary stenosis, or coronary revascularization: coronary artery bypass grafting or percutaneous coronary intervention) and the occurrence of CI. It was found that CHD is significantly associated with the development of na-MCI compared to the general population (OR = 1.93; 95% CI = 1.22–3.06), but not with a-MCI (OR = 0.94; 95% CI = 0.69–1.28). It was also shown that patients with CHD perform worse in neuropsychological tests measuring psychomotor speed, attention and the overall domain score for executive functions, but not with measures of memory. The results of the above studies indicate a relationship between vascular pathology and na-MCI, suggesting that na-MCI can be a prodromal for VaD [13].

A Danish paper by Sundbøll et al., based on a nationwide population-based cohort study involving 314,911 patients with a first-time MI, showed a significantly higher risk of VaD (aHR, 1.35; 95% CI, 1.28–1.43). An additional increase in risk was observed in patients who had had a stroke during the first year after MI (aHR, 4.48; 95% CI, 3.29–6.12). The risk for AD was marginally lower (aHR, 0.92; 95% CI, 0.88–0.95). The results obtained showed no significant association with all-cause dementia compared with the general population cohort (aHR, 1.01; 95%CI, 0.98–1.03) [15].

In the recently published ICON-1 study, 298 elderly patients (mean age 80.5 ± 4.8 years) with NST-ACS undergoing invasive care were evaluated for CI during index hospitalization, and subsequently one year after the vascular incident using the Montreal Cognitive Assessment Scale (MoCA). At the baseline of the study, 48% (n = 130) of the participants had undiagnosed cognitive deficits (MoCA score < 26 points), while one year later 35.1% (n = 74) of the subject developed a cognitive decline (MoCA score drop by ≥2 points). Recurrent MI was associated independently with a worse cognitive outcome [16].

The impact of MI on dementia remains controversial. A study conducted by Brusi et al., did not show any significant evidence of a positive association between them [17]. A similar result was obtained in the Honolulu-Asia Aging Study, in which no association between MI and later CI was demonstrated [18]. In addition, attempts have been made to summarize the divergent data contained in the available literature. A meta-analysis of 10 prospective cohort studies showed that CHD was associated with a 45% increase in the risk of dementia, CI or cognitive decline (OR = 1.45, 95%CI = 1.21 ± 1.74, p < 0.001). Similar results were obtained by analyzing MI and AP separately. However, too little research has been done to perform meta-analyzes for specific types of dementia. On the other hand, a meta-analysis of four case-control and four cross-sectional studies did not yield any significant results, which may be associated with too few studies included in the analysis, as well as heterogeneity of outcomes among studies [19].

## Cognitive impairment – Hypothetical mechanisms in patients with CHD

The exact biological mechanisms underlying the development of CI and dementia in patients with CHD have yet to be explored. It is known, however, that both diseases are associated with common risk factors such as obesity, diabetes, hypertension, hypercholesterolemia, smoking, lack of physical activity, and the presence of the APOE e4 allele [20,21,22,23,24]. However, post-hoc meta-regression analyses did not show a difference between studies that corrected cardiovascular risk factors and those that did not correct them. This suggests that the relationship between CHD and CI cannot be explained solely by cardiovascular burden [19]. Similar conclusions have been drawn following the longitudinal cohort study conducted by Xie et al., which included 7,888 participants without a history of stroke during follow-up. The authors studied cognitive trajectory before and after the CHD episode. As a result of a median follow-up of 12 years, patients with a CHD episode were isolated from other study participants. Among them, 254 participants had had an MI, while 286 were diagnosed with AP. It was observed that patients after CHD had accelerated cognitive decline. A similar relationship was not demonstrated before the event, although they had a greater burden of risk factors. In the same study, comparing MI and AP separately, it was shown that MI was associated with a significantly faster rate of memory loss than AP (p = 0.045) [25]. When analyzing the available literature, it is worth noting that only a few studies address the problem of assessing the cognitive functioning of patients before and after a coronary incident. Also, this is not a common practice in standard clinical procedure. However, it can be expected that due to the increase in CI prevalence and dementia in the general population, as well as the improvement of social awareness, cognitive screening will become more popular.

In a prospective population-based cohort study, Volonghi et al., attempted to match patients for vascular risk factors in order to assess the direct effect of CHD on cognitive function. Three groups of patients were distinguished: after ACS (defined as STEMI, NSTEMI and unstable angina), TIA, and minor stroke (NIHSS ≤ 3). During the follow-up, patients’ cognitive status was assessed twice: after one year (MMSE and TICSm) and after five years (MMSE and MoCa). The results of the study showed that at one year of observation, people after ACS had worse results in MMSE compared to patients after TIA (p < 0.0001) and minor stroke (p = 0.05), and were also more likely to have moderate and severe CI (MMSE < 24) compared to those with TIA OR = 2.14 (95% CI 1.11 –4.13). Similar relationships were observed after five years. ACS was associated with worse MoCA score compared to TIA OR = 2.16 (95% CI 1.01–4.61). It is also significant that despite the selection of study participants for risk factors, survivors of ACS smoked cigarettes more often, while patients after a cerebrovascular incident were more likely to suffer from hypertension and atrial fibrillation. It has also been observed that memory and language function are more often impaired after ACS, whereas executive functions are lower after TIA and stroke [26]. The profile of impaired cognitive domains in patients after ACS suggests that underlying these disorders there may be mechanisms in conjunction with AD, for which the onset with selective memory impairment is also characteristic. This is consistent with the results of recent research, in which the role of cardiovascular disease in the etiopathogenesis of neurodegeneration is increasingly emphasize CI, the hypothesis regarding the role of microRNA is gaining in importance. It is a single-stranded noncoding RNA sequence that typically consists of 22 nucleotides and is involved in the post-transcriptional regulation of gene expression through sequence-specific hybridization to the 3’ untranslated region of mRNAs. They degrade the target mRNA and consequently inhibit the expression of a target protein [27,28]. MiRNAs are mainly located intracellularly, but they can also be found in many body fluids such as plasma, urine, saliva, tears and breast milk [29]. Many show tissue specificity, which would make them potential biomarkers of pathologies occurring in various tissues and organs [30,31]. In the context of this paper, the most interesting fact is the presence of miRNA in exosomes, demonstrated in various studies. These are small membrane vesicles, released by numerous cell types into the extracellular matrix, which play an important role in the process of intercellular communication, transporting not only miRNAs, but also proteins and other nucleic acids [32,33]. Available studies show that patients have a change in blood levels of miRNA after MI. Yuri D’Alessandra et al., noted that miRNA-1, 133a, 133b and 499-5p plasma levels are then increased, both in humans and mice, while miRNA-122 and 375 levels are reduced only in humans. For research purposes, animal models are being increasingly used to reflect human disorders. Whilst looking for a potential path connecting the heart to the brain, Ma JC et al., used a transgenic (Tg) mouse model of cardiac-specific over-expression of microRNA-1-2 (miR-1-2), which showed elevated levels of mi-RNA-1 not only in the heart, but also in the blood and hippocampus of Tg mice compared with age-matched wild-type (WT) mice. Six-month-old Tg mice have also been shown to exhibit CI in the Morris Water Maze test compared with age-matched WT mice. This can be prevented by hippocampal stereotaxic injection of an anti-miR-1 oligonucleotide fragment carried by a lentivirus vector [34]. In the course of further studies using the mouse model of MI obtained by ligation of the left coronary artery (LCA), the same group of researchers showed that overexpression of miRNA-1 in the hippocampus, responsible for damage to neuronal microtubules, may be a direct consequence of MI in a mechanism independent of chronic brain hypoperfusion. and in connection with mi-RNA-1 transport from the heart to the brain through exosomes. Evidence from animal model studies suggests the important role of miRNA in the pathophysiology of cognitive deficits after MI, and also highlights the possibility of their use as a potential therapeutic target [35].

## Cognitive dysfunctions, diagnosis criteria, evidence gaps, clinical doubts

Most dementia diagnoses are made retrospectively based on symptoms reported by the patients or observers. This is associated with the risk of hyperdiagnosis of VaD in patients after MI due to the presence of vascular diseases. When analyzing data from ‘brain banks’ and neuropathological studies, it is worth mentioning that pure forms of VaD are relatively rare [36,37]. Section tests are increasingly recognizing mixed forms in which dementia is the result of the coexistence of primary degenerative (usually AD) and vascular pathologies [38]. Some researchers believe that this is the most common form of dementia [39]. In view of the above, the question arises whether vascular changes overlap with the primary existing AD by triggering its onset, or are they a direct cause of the development of degenerative changes in the brain? The results of recent studies also support the vascular origin of neurodegeneration. They show that statins and hypertension treatment may reduce the incidence of AD [40]. At the same time, this may indicate the pleiotropic effects of these drugs (anti-inflammatory, antiproliferative, antithrombotic, antioxidant), which go beyond the effect on the vascular wall and affect other compartments, including cells of the central nervous system. The relationship between statins and plasma lipoproteins and cognitive function is a complex issue that is not yet fully understood. We know that plasma lipoproteins and cholesterol cannot cross the blood-cerebrospinal fluid barrier (BCSFB), and thus do not affect the functioning of the central nervous system (CNS). An exception is apolipoprotein A1 (ApoA-I). This is the main component of plasma HDL, which can cross the BCSFB. Studies have shown that increased levels of HDL, contributed by statins, protect against cognitive impairment and neurodegeneration. This may explain one of the mechanisms by which these drugs affect cognitive function [41].

A recently published systematic review noted that the overall CI prevalence rate post-ACS is between 9 and 85% in both the short and the long-term [42]. Such a wide range of values may result from the lack of clear definition of CI as well as from the heterogeneity of the conducted studies, which have used various cognitive screening tools with diverse levels of sensitivity and cut-off scores and diverse timepoints measured. Consequently, it is difficult to perform a meta-analysis of the available studies and draw any conclusions.

Among the most common cognitive domains which are impaired after ACS are verbal fluency, language and memory. People affected by these deficits may have difficulty following medical recommendations regarding pharmacotherapy and lifestyle modification (compliance and adherence). In consequence, they will not achieve the therapeutic goals prescribed in the guidelines. That is why the assessment of cognitive function in patients after MI is so important. The lack of early diagnosis of cognitive deficits can lead to serious clinical implications, such as an increase in the number of hospitalizations and mortality. With the knowledge that cardiovascular risk factors are also risk factors for mental deterioration, the preventive actions of clinicians should be directed towards their regular detection and better therapeutic outcomes in the future. Antihypertensive and anticoagulant therapy as well as the use of statins are among the methods of treatment with a proven impact on cognitive functioning [43,44]. Additional interventions supporting pharmacotherapy are regular physical exercise (six months) and cognitive training [5].

## Conclusions

To sum up, cognitive dysfunction is associated with a worse prognosis for patients after ACS, and can also adversely affect further course of treatment. The use of neuropsychological tests should be a routine practice in the care of patients after MI. Further research is needed to clarify the mechanisms underlying CI in patients after MI, so as to be able to devise effective prevention and treatment methods, thus hindering the development of dementia.
